# The Bichloride of Gold Industry

**Published:** 1892-04

**Authors:** 


### THE BICHLORIDE OF GOLD INDUSTRY.

The Keeley treatment is said to have been originated by a chemist in Chicago named Hargrave. A writer in the *Western Medical Review* says that Hargrave denies the presence of gold in the nostrum. According to the originator, it does contain the red bark of cinchona and tincture of nux vomica combined with other bitter tonics for the alcoholics, and the same with the addition of Jamaica dogwood for the opium habitués. By the way, the graduates of the Keeley Institute throughout the country held a convention at Dwight, Illinois, in February. There were three hundred of them, and according to the *Banner of Gold*, the official organ of the Keeley humbug, there was not the taint of alcohol in the air from the breath of one of the redeemed.

Resolutions of mutual admiration were passed, and the notorious Dr. Keeley, their "benefactor and saviour," was deified.

We regret to note that among the cured the mortality from chronic drunkenness has been rather large of late. Following the lead of Col. Mines, who described so beautifully and gratefully what the treatment had accomplished in his case and then died of delirium tremens before his article had left the printer, there have been many since who have paid a parting tribute to their redeemer and died gloriously inebriate. But the swindling process at Dwight goes swimmingly on to the tune, it is said, of $3,000 per day.

Verily, the people like to be humbugged; and the more they pay for it, it seems, the better they like it.

